# Supplementary material for: A Fluorogenic Assay: Analysis of Chemical Modification of Lysine and Arginine to Control Proteolytic Activity of Trypsin
Source: Molecules. 2021 Mar 31;26(7):1975. doi: 10.3390/molecules26071975 (PMC8037237; doi:10.3390/molecules26071975)
Supplement: Supplementary file 1 [file molecules-26-01975-s001.pdf]

SUPPLEMENTARY MATERIAL

A Fluorogenic Assay: Analysis of Chemical Modification of Lysine and Arginine to Control Proteolytic Activity of Trypsin

Kunal N. More [1,†], Tae-Hwan Lim[1,†], Julie Kang [1], and Dong-Jo Chang [1,*]

[1] College of Pharmacy and Research Institute of Life and Pharmaceutical Sciences, Sunchon National University, 255 Jungang-ro, Suncheon 57922, Republic of Korea

[†] These authors contributed equally to this work.

Corresponding author:

*Dong-Jo Chang, Associate Professor
College of Pharmacy, Sunchon National University
Suncheon 57922, Republic of Korea
Tel.: +82-61-750-3765, Fax: +82-61-750-3708
E-mail: djchang@scnu.ac.kr

Table of Contents

A. Synthesis Experimental Procedures······S3-S12

B. Semilogarithmic Plots from Concentration-response Curves of Chemical Modification of Amino Acids······S13-S21

A. Synthesis Experimental Procedures

General information

All starting materials and reagents were purchased from Sigma-Aldrich Chemical Co., St. Louis, MO, USA; Tokyo Chemical Industries, Tokyo, Japan; Daejung Chemicals, Siheung-si, Korea; and Alfa Aesar, Ward Hill, MA, USA, and were used without any further purification. Solvents were purified using a PureSolv Micro Multi Unit solvent purification system obtained from Inert Technology (Amesbury, MA, USA) and were used under a dry nitrogen atmosphere. The progress of reactions was assessed by thin-layer chromatography on silica gel plates (Kiesegel $60F_{254}$; Merck; Darmstadt, Germany), and the synthesized compounds were purified by flash column chromatography using silica gel (ZEOprep 60; 40–63 μm). ^{1}H NMR and ^{13}C spectra were measured with a JEOL JNM-ECZ400s/L1 (400 MHz) spectrometer (Tokyo, Japan), using $CDCl_3$ or DMSO-d_6 as the NMR solvent (Cambridge Isotope Laboratories, Tewksbury, MA, USA). ^{1}H NMR chemical shifts are expressed in terms of parts per million (ppm) based on the chemical shift of tetramethylsilane (δ = 0 ppm) in $CDCl_3$ as an internal standard. The chemical shifts in ^{13}C NMR are reported in ppm relative to the centerline of the triplet at 77.0 ppm observed for $CDCl_3$ or 39.5 ppm for DMSO-d_6. The coupling constant J in ^{1}H NMR is reported in hertz (Hz). Fluorogenic assays were performed using a Synergy™ H1 microplate reader from BioTek Instruments (Winooski, VT, USA).. Trypsin from porcine pancreas was purchased from Sigma-Aldrich Chemical Co., St. Louis, MO, USA. The enzyme solution was freshly prepared prior to performing assays by dissolving lyophilized trypsin powder in assay buffer (50 mM HEPES, pH 8.3). Stock solutions of the fluorogenic peptide substrate and amino acid blockers were prepared in DMSO.

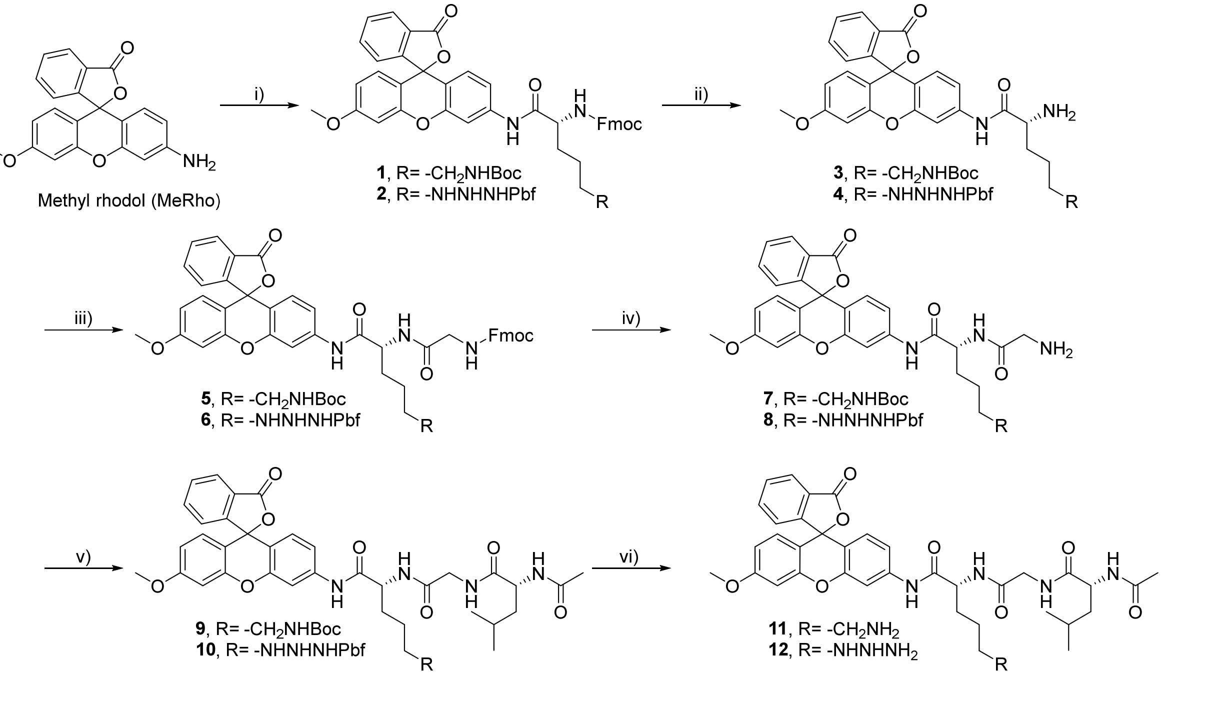

Scheme 1. Synthesis of tripeptide fluorogenic substrates MeRho-Lys-Gly-Leu(Ac) and MeRho-Arg-Gly-Leu(Ac): i) Fmoc-Lys(Boc)-OH, EEDQ, Chloroform, rt, or Fmoc-Arg(Pbf)-OH, EDC, DMAP, CH_2Cl_2, rt, **1** = 82%, **2** = 64%; ii) $(CH_2)_5NH$, CH_3CN, rt, **3** = 96%, **4** = 96%; iii) Fmoc-Gly-OH, DIC, HOBt, CH_2Cl_2, rt, **5** = 80%, **6** = 73% ; iv) $(CH_2)_5NH$, CH_3CN, rt, **7** = 95%, **8** = 97%; v) Ac-Leu-OH, DIC, HOBt, CH_2Cl_2, rt, **9** = 76%, **10** = 76%; vi) 10 % TFA, CH_2Cl_2, rt, **11** = 42%, **12** = 45%.

General Synthetic Procedures

A. General Procedure A: Amide Coupling

To a solution of aniline or amine (1.0 eq.) and AA (1.2 eq.) in CH_2Cl_2,we added DIC (1.2 eq.) and HOBt (1.2 eq.). The reaction mixture was stirred at room temperature for 2 h, and on completion of the reaction, the reaction solvent was evaporated. The resulting solid was purified by column chromatography to obtain the desired product.

B. General Procedure B: Fmoc Deprotection

To a solution of Fmoc-protected compound (1.0 eq.), we added piperidine (1.2 eq.) in anhydrous CH_3CN (395 eq.). The reaction mixture was stirred at room temperature for 2 h, and on completion of the reaction, the reaction solvent was evaporated. The resulting residue was purified by column chromatography to obtain the desired Fmoc-deprotected product.

C. General Procedure C: Acid-labile Group Deprotection (Boc/Pbf)

To a solution of Boc/Pbf-protected compound (1.0 eq.), we added TFA (10% solvent) in anhydrous CH_2Cl_2. The reaction mixture was stirred at room temperature for 2 h, and on completion of the reaction, the pH of the reaction mixture was increased with 1N NaOH. The compound was extracted using an organic solvent mixture (DCM: MeOH, 90:10), and the organic layer was dried over Na_2SO_4 and filtered, with the resulting filtrate being evaporated under vacuum. The resulting residue was purified by column chromatography to obtain the desired fluorogenic peptide substrate.

1. (9*H*-fluoren-9-yl)methyl tert-butyl ((5R)-6-((3'-methoxy-3-oxo-3*H*-spiro[isobenzofuran-1,9'-xanthen]-6'-yl)amino)-6-oxohexane-1,5-diyl)dicarbamate (**1**)

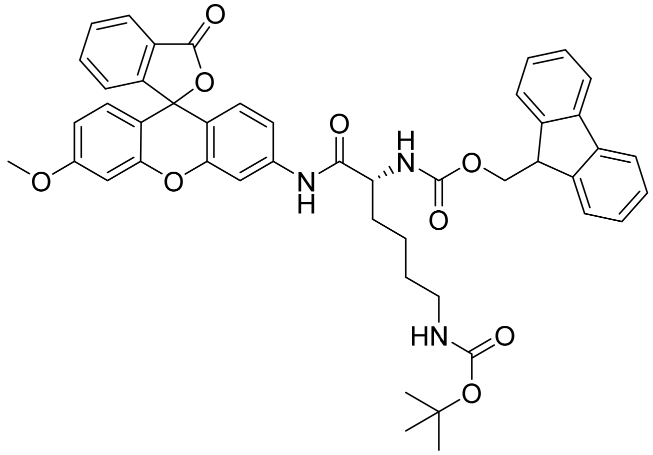

To a solution of methoxy rhodol (MeRho) (100 mg, 0.29 mmol) and Fmoc-Lys(Boc)-OH (163 mg, 0.35 mmol) in Chloroform (8 mL) was added EEDQ (86 mg, 0.35 mmol). The reaction mixture was stirred at room temperature (rt) for 1.5 h. After the completion of the reaction, the reaction solvent was evaporated and the resulting solid was purified by column chromatography to afford the desired compound **1** in 82 % yield (190 mg); ^{1}H-NMR (400 MHz, DMSO-d_6) δ 10.32 (s, 1H), 7.98 (d, J = 7.8 Hz,

1H), 7.85 (d, J = 7.3 Hz, 3H), 7.76 (t, J = 7.5 Hz, 1H), 7.72-7.65 (m, 4H), 7.38 (t, J = 7.3 Hz, 2H), 7.31-7.27 (m, 2H), 7.23 (dd, J = 7.8, 0.9 Hz, 1H), 7.17-7.13 (m, 1H), 6.94 (d, J = 2.3 Hz, 1H), 6.75-6.69 (m, 2H), 6.67 (d, J = 2.3 Hz, 1H), 6.64 (d, J = 9.1 Hz, 1H), 4.24 (d, J = 7.8 Hz, 2H), 4.18 (t, J = 7.1 Hz, 1H), 4.08 (q, J = 7.3 Hz, 1H), 3.78 (s, 3H), 3.29 (s, 2H), 2.87 (br, 2H), 1.61 (br, 2H), 1.34 (s, 2H), 1.30 (s, 9H); 13C-NMR (100 MHz, DMSO-*d*6) δ 172.4, 169.2, 161.6, 156.6, 156.1, 153.1, 152.3, 151.4, 144.4, 144.3, 141.6, 141.3, 136.3, 130.8, 129.5, 129.0, 128.2, 127.6, 126.3, 125.9, 125.3, 124.5, 120.6, 115.9, 113.8, 112.7, 111.3, 106.8, 101.4, 82.6, 77.9, 66.2, 56.2, 56.1, 55.4, 47.2, 31.9, 29.8, 28.8, 23.5; HRMS (ESI+): m/z Calcd for $C_{47}H_{46}N_3O_9$ [M+H]+: 796.3234, Found: 796.3229.

2. (9*H*-fluoren-9-yl)methyl ((2*R*)-1-((3'-methoxy-3-oxo-3*H*-spiro[isobenzofuran-1,9'-xanthen]-6'-yl)amino)-1-oxo-5-(3-((2,2,4,6,7-pentamethyl-2,3-dihydrobenzofuran-5-yl)sulfonyl)guanidino)pentan-2-yl)carbamate (**2**)

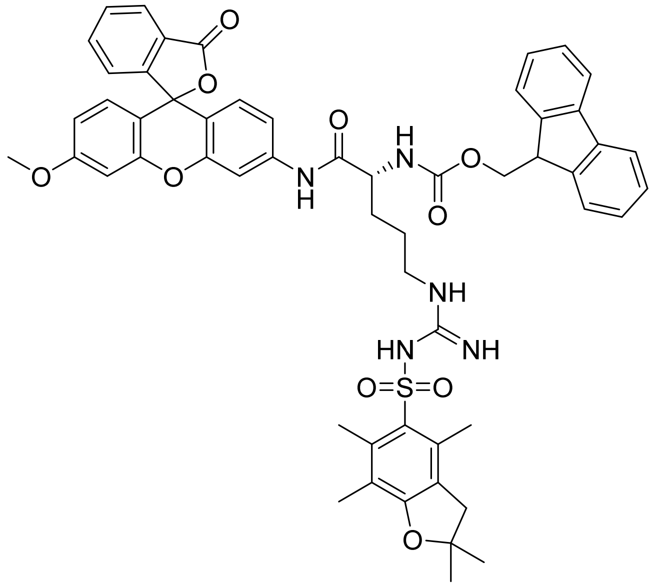

To a solution of methoxy rhodol (MeRho) (50 mg, 0.15 mmol) and Fmoc-Arg(Pbf)-OH (122 mg, 0.19 mmol) in CH_2Cl_2 (10 mL) was added EDC (111 mg, 0.58 mmol) and DMAP (26.57 mg, 0.22 mmol). The reaction mixture was stirred at room temperature (rt) for 1 h. After the completion of the reaction, the reaction solvent was evaporated and the resulting solid was purified by column chromatography to afford the desired compound **2** in 64 % yield (90 mg); ^{1}H-NMR (400 MHz, DMSO-*d*6) δ 10.36 (s, 1H), 8.01 (d, J = 7.8 Hz, 1H), 7.88 (d, J = 7.3 Hz, 3H), 7.79 (t, J = 7.5 Hz, 1H), 7.72 (t, J = 7.1 Hz, 4H), 7.40 (t, J = 7.3 Hz, 2H), 7.31 (t, J = 7.3 Hz, 2H), 7.26 (d, J = 7.3 Hz, 1H), 7.19-7.16 (m, 1H), 6.98 (d, J = 2.3 Hz, 1H), 6.74 (d, J = 9.1 Hz, 2H), 6.70 (d, J = 2.3 Hz, 1H), 6.67 (d, J = 8.7 Hz, 1H), 6.37 (s, 1H), 4.27 (d, J = 6.9 Hz, 2H), 4.21 (t, J = 7.1 Hz, 1H), 4.13 (q, J = 7.3 Hz, 1H), 3.81 (s, 3H), 3.06 (br, 2H), 2.91 (s, 2H), 2.45 (s, 3H), 2.38 (s, 3H), 1.95 (s, 3H), 1.67-1.59 (m, 2H), 1.51-1.42 (m, 2H), 1.36 (s, 6H); 13C-NMR (100 MHz, DMSO-*d*6) δ 172.1, 169.2, 161.7, 158.0, 156.6, 153.1, 152.3, 151.4, 144.4, 144.3, 141.5, 141.3, 137.8, 136.3, 132.0, 130.8, 129.5, 129.0, 128.2, 127.6, 126.3, 125.8, 125.3, 124.8, 124.5, 120.6, 116.8, 116.0, 113.9, 112.7, 111.3, 106.8, 101.4, 86.8, 82.6, 66.2, 63.3, 56.2, 55.7, 55.4, 47.2, 43.0, 28.8, 19.4, 18.1, 12.8; HRMS (ESI+): m/z Calcd for $C_{55}H_{54}N_5O_{10}S$ [M+H]+: 976.3591, Found: 976.3586.

3. *Tert*-butyl (5-amino-6-((3'-methoxy-3-oxo-3*H*-spiro[isobenzofuran-1,9'-xanthen]-6'-yl)amino)-6-oxohexyl)carbamate (**3**)

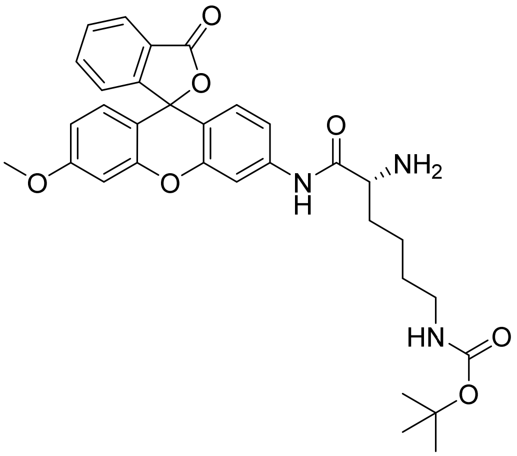

Compound **3** (174 mg) was synthesized in 96% yield via the deprotection of Fmoc from compound **1** (250 mg, 0.31 mmol) using piperidine (32 mg, 0.38 mmol) in anhydrous CH_3CN (6.48 mL) according to general procedure B; 1H-NMR (400 MHz, $CDCl_3$) δ 9.74 (s, 1H), 8.00 (d, J = 7.3 Hz, 1H), 7.82 (dd, J = 5.7, 1.6 Hz, 1H), 7.64 (t, J = 7.3 Hz, 1H), 7.59 (t, J = 7.3 Hz, 1H), 7.12 (d, J = 7.3 Hz, 1H), 7.06 (t, J = 6.4 Hz, 1H), 6.75 (d, J = 2.3 Hz, 1H), 6.70 (d, J = 8.7 Hz, 1H), 6.67 (d, J = 8.7 Hz, 1H), 6.59 (dd, J = 8.7, 2.3 Hz, 1H), 4.61 (s, 1H), 3.82 (s, 3H), 3.54 (s, 1H), 3.10 (s , 2H), 1.94 (br, 1H), 1.65-1.56 (m, 1H), 1.48 (br, 3H), 1.41 (s, 9H); 13C-NMR (100 MHz, $CDCl_3$) δ 169.6, 161.5, 156.3, 153.3, 152.5, 151.9, 139.8, 135.1, 129.8, 129.0, 128.6, 126.7, 125.1, 124.0, 115.3, 114.5, 111.8, 111.0, 107.5, 100.9, 83.0, 79.3, 55.7, 55.2, 53.5, 40.0, 29.8, 28.5, 22.6; HRMS (ESI$^+$): m/z Calcd for $C_{32}H_{36}N_3O_7$ [M+H]$^+$: 574.2553, Found: 574.2546.

4. (2*R*)-2-Amino-*N*-(3'-methoxy-3-oxo-3*H*-spiro[isobenzofuran-1,9'-xanthen]-6'-yl)-5-(3-((2,2,4,6,7-pentamethyl-2,3-dihydrobenzofuran-5-yl)sulfonyl)guanidino)pentanamide (**4**)

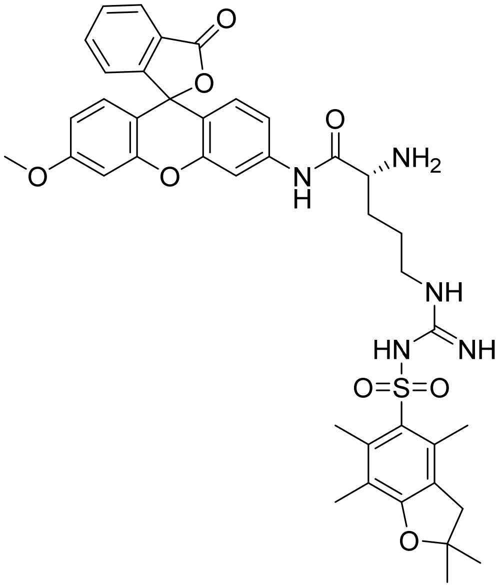

Compound **4** (90 mg) was synthesized in 96% yield via the deprotection of Fmoc from compound **2** (122 mg, 0.13 mmol) using piperidine (12.77 mg, 0.15 mmol) in anhydrous CH_3CN (2.6 mL) according to general procedure B; ^{1}H-NMR (400 MHz, DMSO-d_6) δ 7.99 (d, J = 7.8 Hz, 1H), 7.90 (dd, J = 6.4, 1.8 Hz, 1H), 7.76 (t, J = 7.5 Hz, 1H), 7.69 (t, J = 7.3 Hz, 1H), 7.23 (d, J = 7.3 Hz, 1H), 7.20-7.16 (m, 1H), 6.95 (d, J = 2.7 Hz, 1H), 6.71-6.63 (m, 4H), 6.33 (s, 1H), 3.78 (s, 3H), 3.01 (d, J = 5.5 Hz, 2H), 2.89 (s, 2H), 2.42 (s, 3H), 2.36 (s, 3H), 1.93 (s, 3H), 1.57-1.55 (m, 1H), 1.49-1.44 (m, 1H), 1.42-1.38 (m, 2H), 1.34 (s, 6H); 13C-NMR (100 MHz, DMSO-d_6) δ 175.5, 169.2, 161.6, 157.9, 156.6, 153.1, 152.3, 151.4, 141.6, 137.8, 136.3, 132.0, 130.8, 129.5, 128.9, 126.3, 125.3, 124.8, 124.5, 116.8, 116.0, 113.7, 112.6, 111.3, 106.7, 101.4, 86.8, 82.7, 56.2, 55.9, 43.0, 28.8, 19.5, 18.1, 12.8; HRMS (ESI$^+$): m/z Calcd for $C_{40}H_{44}N_5O_8S$ [M+H]$^+$: 754.2911, Found: 754.2908.

5. (9*H*-fluoren-9-yl)methyl (2-(((2*R*)-6-((*tert*-butoxycarbonyl)amino)-1-((3'-methoxy-3-oxo-3*H*-spiro[isobenzofuran-1,9'-xanthen]-6'-yl)amino)-1-oxohexan-2-yl)amino)-2-oxoethyl)carbamate (**5**)

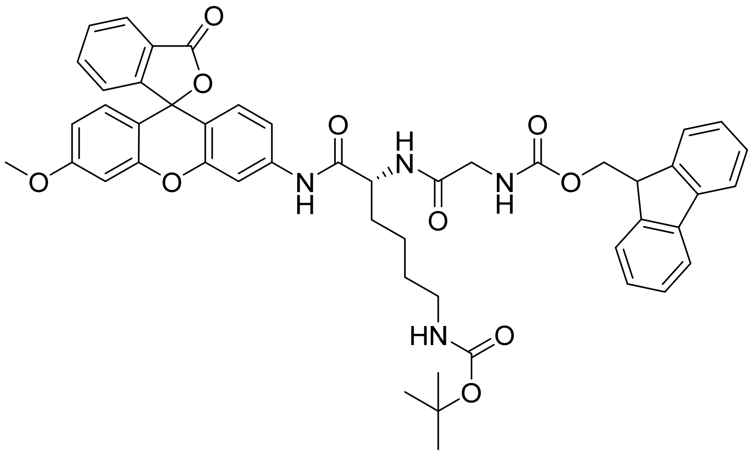

Compound **5** (130 mg) was synthesized in 80% yield via the amide coupling of compound **3** (109 mg, 0.19 mmol) with Fmoc-Gly-OH (62.14 mg, 0.21 mmol), using DIC (28.78 mg, 0.23 mmol) and HOBT (30.80 mg, 0.23 mmol) according to general procedure A. ^{1}H-NMR (400 MHz, DMSO-d_6) δ 10.24 (s, 1H), 8.13 (d, J = 7.8 Hz, 1H), 7.99 (d, J = 7.8 Hz, 1H), 7.83 (t, J = 7.1 Hz, 3H), 7.76 (t, J = 7.3 Hz, 1H), 7.70 (d, J = 7.3 Hz, 1H), 7.66 (d, J = 7.8 Hz, 2H), 7.50 (t, J = 5.9 Hz, 1H), 7.38-7.34 (m, 2H), 7.27 (td, J = 7.4, 2.1 Hz, 2H), 7.22 (d, J = 7.3 Hz, 1H), 7.17 (d, J = 10.1 Hz, 1H), 6.92 (d, J = 1.8 Hz, 1H), 6.69 (dd, J = 9.1, 2.3 Hz, 3H), 6.64 (d, J = 8.7 Hz, 1H), 4.35 (q, J = 7.0 Hz, 1H), 4.24 (d, J = 5.9 Hz, 2H), 4.18 (t, J = 7.1 Hz, 1H), 3.78 (s, 3H), 3.65 (d, J = 5.9 Hz, 2H), 2.84 (d, J = 5.9 Hz, 2H), 1.68-1.64 (m, 1H), 1.59-1.51 (m, 1H), 1.33-1.32 (m, 3H), 1.30 (s, 9H); 13C-NMR (100 MHz, DMSO-d_6) δ 171.8, 169.7, 169.2, 161.6, 157.1, 156.1, 153.1, 152.3, 151.4, 144.4, 141.5, 141.2, 136.3, 130.8, 129.5, 129.0, 128.1, 127.6, 126.3, 125.8, 125.3, 124.5, 120.6, 116.0, 113.9, 112.6, 111.3, 106.9, 101.4, 82.6, 77.8, 66.3, 56.2, 54.0, 47.2, 43.8, 32.3, 29.8, 28.8, 23.2; HRMS (ESI$^+$): m/z Calcd for $C_{49}H_{49}N_4O_{10}$ [M+H]$^+$: 853.3449, Found: 853.3448.

6. (9*H*-fluoren-9-yl)methyl (2-(((2*R*)-1-((3'-methoxy-3-oxo-3*H*-spiro[isobenzofuran-1,9'-xanthen]-6'-yl)amino)-1-oxo-5-(3-((2,2,4,6,7-pentamethyl-2,3-dihydrobenzofuran-5-yl)sulfonyl)guanidino)pentan-2-yl)amino)-2-oxoethyl)carbamate (**6**)

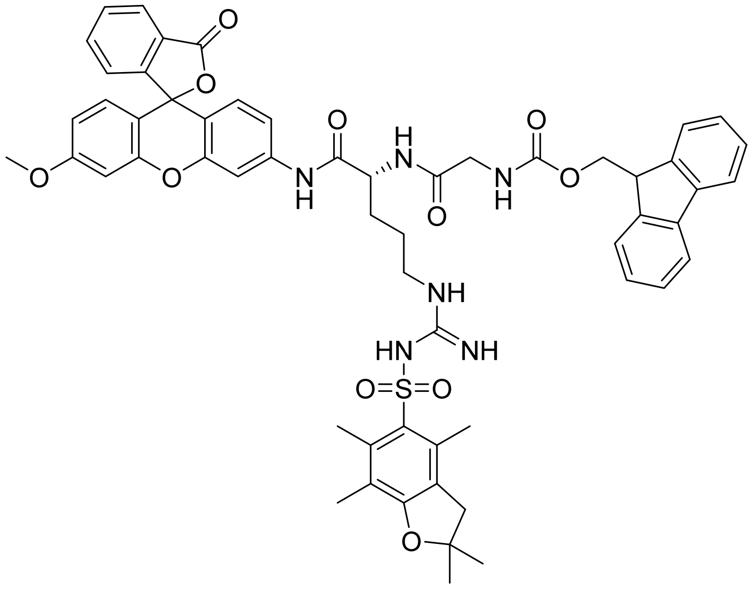

Compound **6** (90 mg) was synthesized in 73% yield via the amide coupling of compound **4** (90 mg, 0.12 mmol) with Fmoc-Gly-OH (22.27 mg, 0.13 mmol), using DIC (17.71 mg, 0.14 mmol) and HOBT (18.97 mg, 0.14 mmol) according to general procedure A. ^{1}H-NMR (400 MHz, DMSO-d_6) δ 10.29 (s, 1H), 8.20 (d, J = 7.8 Hz, 1H), 7.99 (d, J = 7.8 Hz, 1H), 7.87 (d, J = 6.8 Hz, 3H), 7.76 (t, J = 7.3 Hz, 1H), 7.71 (d, J = 7.3 Hz, 1H), 7.66 (d, J = 7.3 Hz, 2H), 7.53 (t, J = 6.2 Hz, 1H), 7.38-7.34 (m, 2H), 7.28-7.22 (m, 3H), 7.17 (d, J = 8.7 Hz, 1H), 6.93 (d, J = 1.8 Hz, 1H), 6.71 (d, J = 7.8 Hz, 1H), 6.68 (d, J = 2.3 Hz, 1H), 6.64 (d, J = 8.7 Hz, 1H), 6.33 (s, 1H), 4.38 (q, J = 7.2 Hz, 1H), 4.24 (d, J = 6.4 Hz, 2H), 4.18 (t, J = 6.9 Hz, 1H), 3.78 (s, 3H), 3.64 (d, J = 5.9 Hz, 2H), 3.01 (s, 2H), 2.86 (s, 2H), 2.41 (s, 3H), 2.34 (s, 3H), 1.92 (s, 3H), 1.70-1.65 (m, 1H), 1.56-1.50 (m, 1H), 1.46-1.36 (m, 2H), 1.33 (s, 6H); 13C-NMR (100 MHz, DMSO-d_6) δ 171.6, 169.7, 161.6, 158.0, 157.1, 156.6, 153.1, 152.3, 151.4, 144.3, 141.4, 141.2, 137.8, 136.3, 132.0, 130.8, 129.5, 129.0, 128.1, 127.6, 126.3, 125.8, 125.3, 124.8, 124.5, 120.6, 116.8, 116.0, 114.0, 112.7, 111.2, 101.4, 86.8, 82.6, 66.3, 56.2, 47.1, 43.8, 42.9, 28.8, 19.5, 18.1, 12.8; HRMS (ESI$^+$): m/z Calcd for $C_{57}H_{57}N_6O_{11}S$ [M+H]$^+$: 1033.3806, Found: 1033.3805.

7. *Tert*-butyl ((5*R*)-5-(2-aminoacetamido)-6-((3'-methoxy-3-oxo-3*H*-spiro[isobenzofuran-1,9'-xanthen]-6'-yl)amino)-6-oxohexyl)carbamate (**7**)

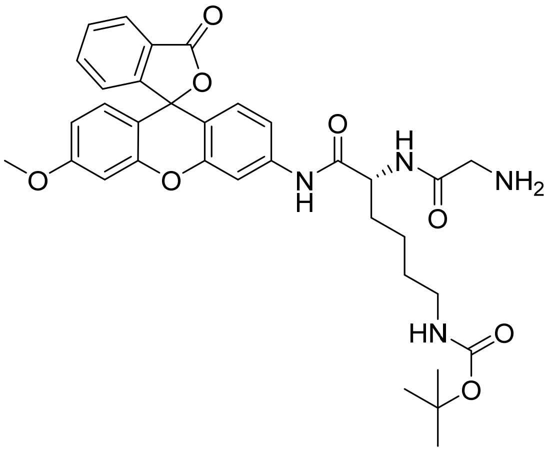

Compound **7** was synthesized from compound **5** (30 mg, 0.035 mmol) via the deprotection of Fmoc using piperidine (3.59 mg, 0.042 mmol) in anhydrous CH_3CN (726 μL), according to general procedure B. The residue was purified by flash column chromatography on silica gel to obtain compound **7** (21 mg) in 95 % yield. ^{1}H-NMR (400 MHz, DMSO-d_6) δ 10.36 (d, J = 2.7 Hz, 1H), 8.08 (s, 1H), 7.98 (d, J = 7.8 Hz, 1H), 7.81 (dd, J = 14.6, 1.8 Hz, 1H), 7.76 (t, J = 7.1 Hz, 1H), 7.69 (t, J = 7.1 Hz, 1H), 7.23 (dd, J = 7.8, 2.7 Hz, 1H), 7.19-7.14 (m, 1H), 6.94 (d, J = 2.3 Hz, 1H), 6.70 (d, J = 8.7 Hz, 2H), 6.67 (d, J = 2.3 Hz, 1H), 6.64 (d, J = 9.1 Hz, 1H), 4.40 (s, 1H), 3.78 (s, 3H), 3.35 (s, 1H), 3.11 (s, 2H), 2.84 (q, J = 6.1 Hz, 2H), 1.68-1.63 (m, 1H), 1.60-1.57 (m, 1H), 1.35-1.31 (m, 3H), 1.30 (s, 9H); 13C-NMR (100 MHz, DMSO-d_6) δ 173.2, 171.8, 169.2, 161.7, 156.1, 153.1, 152.3, 151.4, 141.5, 136.3, 130.8, 129.5, 129.0, 126.3, 125.3, 124.5, 116.0, 113.9, 112.7, 111.3, 106.9, 101.4, 82.6, 77.8, 63.3, 56.2, 55.4, 53.6, 44.9, 32.7, 29.8, 28.8, 23.1; HRMS (ESI$^+$): m/z Calcd for $C_{34}H_{39}N_4O_8$ [M+H]$^+$: 631.2768, Found: 631.2768.

8. (2*R*)-2-(2-aminoacetamido)-*N*-(3'-methoxy-3-oxo-3*H*-spiro[isobenzofuran-1,9'-xanthen]-6'-yl)-5-(3-((2,2,4,6,7-pentamethyl-2,3-dihydrobenzofuran-5-yl)sulfonyl)guanidino)pentanamide (**8**)

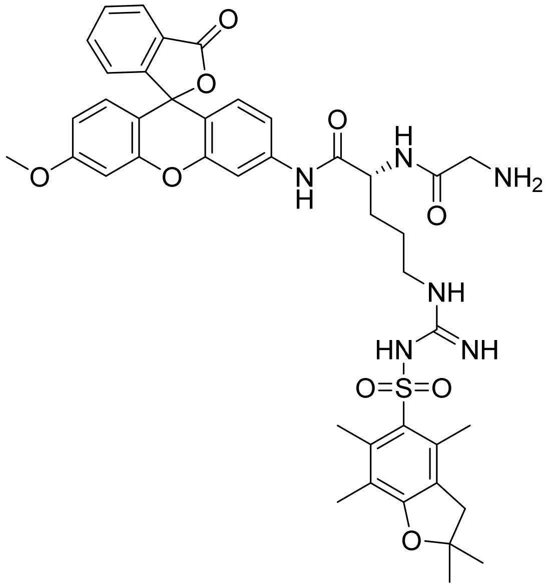

Compound **8** was synthesized from compound **6** (85 mg, 0.082 mmol) via the deprotection of Fmoc using piperidine (8.4 mg, 0.099 mmol) in anhydrous CH_3CN (1.7 mL), according to general procedure B. The residue was purified by flash column chromatography on silica gel to give compound **8** (65 mg) in 97 % yield. ^{1}H-NMR (400 MHz, DMSO-d_6) δ 10.45 (s, 1H), 8.20 (s, 1H), 7.99 (d, J = 7.3 Hz, 1H), 7.82 (dd, J = 8.7, 1.8 Hz, 1H), 7.76 (t, J = 7.3 Hz, 1H), 7.69 (t, J = 7.5 Hz, 1H), 7.23 (dd, J = 7.3, 2.3 Hz, 1H), 7.18 (td, J = 7.8, 1.8 Hz, 1H), 6.95 (d, J = 2.3 Hz, 1H), 6.71 (d, J = 9.1 Hz, 1H), 6.68 (d, J = 2.3 Hz, 1H), 6.64 (d, J = 8.7 Hz, 1H), 6.34 (s, 1H), 4.44 (s, 1H), 3.78 (s, 3H), 3.15 (s, 2H), 3.01 (q, J = 6.1 Hz, 2H), 2.88 (s, 2H), 2.41 (s, 3H), 2.35 (s, 3H), 1.92 (s, 3H), 1.72-1.64 (m, 1H), 1.60-1.49 (m, 1H), 1.44-1.38 (m, 2H), 1.34 (s, 6H); 13C-NMR (100 MHz, DMSO-d_6) δ 171.6, 169.2, 161.6, 158.0, 156.6, 153.1, 152.3, 151.3, 141.4, 137.8, 136.3, 132.0, 130.8, 129.5, 129.1, 126.3, 125.3, 124.8, 124.5, 116.8, 116.0, 114.0, 112.7, 111.2, 106.9, 101.4, 86.8, 82.6, 56.2, 53.2, 44.5, 43.0, 30.4, 28.8, 19.5, 18.1, 12.8; HRMS (ESI$^+$): m/z Calcd for $C_{42}H_{47}N_6O_9S$ [M+H]$^+$: 811.3125, Found: 811.3124.

9. *Tert*-butyl ((5*R*)-5-(2-((*R*)-2-acetamido-4-methylpentanamido)acetamido)-6-((3'-methoxy-3-oxo-3*H*-spiro[isobenzofuran-1,9'-xanthen]-6'-yl)amino)-6-oxohexyl)carbamate (**9**)

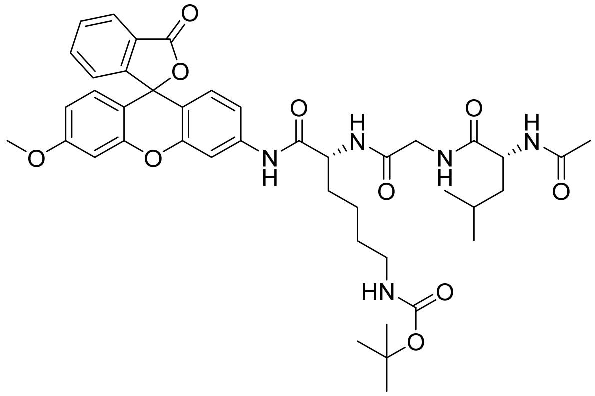

Compound **9** (20 mg) was synthesized in 76% yield via the amide coupling of compound **7** (21 mg, 0.033 mmol) with Ac-Leu-OH (6.34 mg, 0.037 mmol), using DIC (5.04 mg, 0.04 mmol) and HOBT (5.40 mg, 0.04 mmol) according to general procedure A. ^{1}H-NMR (400 MHz, DMSO-d_6) δ 10.16 (d, J = 5.9 Hz, 1H), 8.26 (q, J = 5.9 Hz, 1H), 8.03 (dd, J = 7.5, 2.5 Hz, 1H), 7.97 (t, J = 7.5 Hz, 2H), 7.84 (dd, J = 16.9, 1.8 Hz, 1H), 7.76 (tt, J = 7.5, 1.3 Hz, 1H), 7.69 (t, J = 7.3 Hz, 1H), 7.24-7.18 (m, 2H), 6.93 (d, J = 2.7 Hz, 1H), 6.71 (d, J = 8.7 Hz, 2H), 6.67 (d, J = 2.7 Hz, 1H), 6.64 (d, J = 8.7 Hz, 1H), 4.30 (q, J = 7.2 Hz, 1H), 4.18 (q, J = 7.5 Hz, 1H), 3.78 (s, 3H), 3.73-3.62 (m, 2H), 2.84 (q, J = 6.3 Hz, 2H), 1.79 (d, J = 9.1 Hz, 3H), 1.68-1.61 (m, 1H), 1.60-1.53 (m, 2H), 1.46-1.36 (m, 2H), 1.36-1.30 (m, 2H), 1.28 (s, 9H), 0.81 (ddd, J = 16.8, 6.5, 1.3 Hz, 6H); 13C-NMR (100 MHz, DMSO-d_6) δ 173.5, 173.4, 171.7, 170.1, 169.5, 169.2, 161.6, 156.1, 153.1, 152.3, 151.3, 141.5, 136.3, 130.8, 129.5, 129.0, 126.3, 125.3, 124.5, 116.0, 113.9, 112.6, 111.3, 106.9, 101.4, 82.6, 77.8, 56.2, 55.4, 54.1, 51.9, 42.6, 32.0, 29.8, 28.8, 24.7, 23.5, 23.3, 23.0, 22.1; HRMS (ESI$^+$): m/z Calcd for $C_{42}H_{52}N_5O_{10}$ [M+H]$^+$: 786.3714, Found: 786.3710.

10. (2*R*)-2-acetamido-*N*-(2-(((2*R*)-1-((3'-methoxy-3-oxo-3*H*-spiro[isobenzofuran-1,9'-xanthen]-6'-yl)amino)-1-oxo-5-(3-((2,2,4,6,7-pentamethyl-2,3-dihydrobenzofuran-5-yl)sulfonyl)guanidino)pentan-2-yl)amino)-2-oxoethyl)-4-methylpentanamide (**10**)

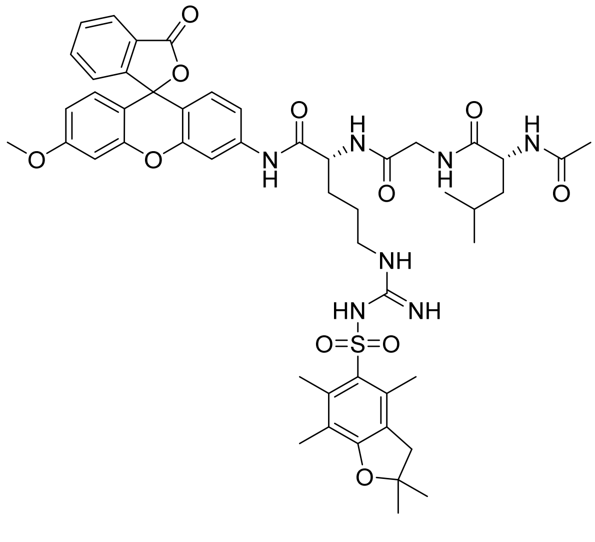

Compound **10** (45 mg) was synthesized in 76% yield via the amide coupling of compound **8** (50 mg, 0.062 mmol) with Ac-Leu-OH (11.75 mg, 0.068 mmol), using DIC (9.39 mg, 0.074 mmol) and HOBT (10.00 mg, 0.074 mmol) according to general procedure A. ^{1}H-NMR (400 MHz, DMSO-d_6) δ 10.20 (d, J = 8.2 Hz, 1H), 8.30 (q, J = 6.1 Hz, 1H), 8.06-8.00 (m, 2H), 7.98 (d, J = 7.8 Hz, 1H), 7.84 (dd, J = 8.7, 1.8 Hz, 1H), 7.76 (t, J = 7.5 Hz, 1H), 7.69 (t, J = 7.3 Hz, 1H), 7.23-7.19 (m, 2H), 6.93 (d, J = 2.3 Hz, 1H), 6.71 (d, J = 9.1 Hz, 1H), 6.68 (d, J = 2.7 Hz, 1H), 6.64 (d, J = 8.7 Hz, 1H), 6.32 (s, 1H), 4.32 (q, J = 6.9 Hz, 1H), 4.17 (q, J = 7.5 Hz, 1H), 3.78 (s, 3H), 3.67 (t, J = 6.6 Hz, 2H), 3.00 (pent, J = 6.3 Hz, 2H), 2.87 (s, 2H), 2.41 (s, 3H), 2.35 (s, 3H), 1.92 (s, 3H), 1.77 (d, J = 9.1 Hz, 3H), 1.71-1.53 (m, 2H), 1.41-1.37 (m, 4H), 1.33 (s, 6H), 0.80 (dd, J = 17.4, 5.9 Hz, 6H); 13C-NMR (100 MHz, DMSO-d_6) δ 171.5, 169.5, 169.2, 161.7, 158.0, 157.3, 156.6, 153.1, 152.3, 151.3, 141.4, 137.8, 136.3, 132.0, 130.8, 129.5, 129.0, 126.3, 125.3, 124.8, 124.6, 124.5, 119.4, 116.8, 116.1, 114.0, 112.7, 111.3, 106.9, 101.4, 86.8, 82.6, 56.2, 53.7, 43.0, 41.2, 29.5, 29.2, 28.8, 24.7, 23.8, 23.4, 22.9, 22.6, 22.2, 19.4, 18.1, 14.5, 12.8; HRMS (ESI$^+$): m/z Calcd for $C_{50}H_{60}N_7O_{11}S$ [M+H]$^+$: 966.4072, Found: 966.4066.

11. (2*R*)-2-(2-((*R*)-2-acetamido-4-methylpentanamido)acetamido)-6-amino-*N*-(3'-methoxy-3-oxo-3H-spiro[isobenzofuran-1,9'-xanthen]-6'-yl)hexanamide (**11**)

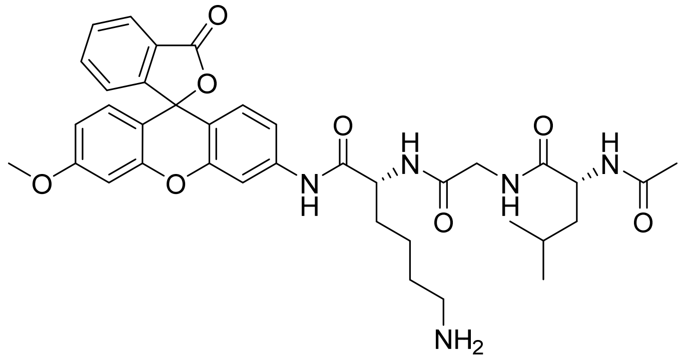

Compound **11** was synthesized from compound **9** (40 mg, 0.050 mmol) via the deprotection of Boc using TFA (10 %, 0.8 mL) in anhydrous CH_2Cl_2 (8 mL), according to general procedure C. The residue was purified by flash column chromatography on silica gel to give compound **11** (16 mg) in 42 % yield. ^{1}H-NMR (400 MHz, DMSO-d_6) δ 10.35 (d, J = 9.6 Hz, 1H), 8.33 (q, J = 5.6 Hz, 1H), 8.10 (dd, J = 7.5, 3.4 Hz, 1H), 8.06 (d, J = 7.3 Hz, 1H), 7.98 (d, J = 7.3 Hz, 1H), 7.86 (dd, J = 7.8, 1.8 Hz, 1H), 7.76 (t, J = 7.5 Hz, 1H), 7.69 (t, J = 7.5 Hz, 2H), 7.28 (dd, J = 8.7, 1.8 Hz, 1H), 7.22 (dd, J = 7.3, 2.7 Hz, 1H), 6.93 (d, J = 2.7 Hz, 1H), 6.70 (d, J = 8.7 Hz, 1H), 6.67 (d, J = 2.3 Hz, 1H), 6.63 (d, J = 9.1 Hz, 1H), 4.33 (q, J = 6.9 Hz, 1H), 4.18 (q, J = 7.3 Hz, 2H), 3.78 (s, 3H), 3.74-3.63 (m, 2H), 2.71 (t, J = 7.5 Hz, 2H), 1.79 (d, J = 9.1 Hz, 3H), 1.63-1.58 (m, 2H), 1.58-1.46 (m, 4H), 1.45-1.38 (m, 1H), 0.81 (ddd, J = 17.2, 6.4, 1.6 Hz, 6H); 13C-NMR (100 MHz, DMSO-d_6) δ 173.5, 173.5, 171.7, 170.2, 169.5, 169.2, 161.6, 158.9, 158.6, 158.3, 158.0, 153.1, 152.3, 151.3, 141.5, 136.3, 132.2, 132.1, 130.8, 129.5, 129.2, 128.9, 126.3, 125.3, 124.5, 122.3, 119.3, 116.3, 116.1, 113.9, 112.6, 111.3, 106.9, 101.4, 82.6, 63.3, 56.2, 54.0, 52.0, 42.7, 41.0, 39.0, 31.6, 29.5, 29.4, 29.2, 27.1, 24.7, 23.5, 23.0, 22.8, 22.6, 22.1; HRMS (ESI$^+$): m/z Calcd for $C_{37}H_{44}N_5O_8$ [M+H]$^+$: 686.3190, Found: 686.3187.

12. (2*R*)-2-Acetamido-*N*-(2-(((2*R*)-5-guanidino-1-((3'-methoxy-3-oxo-3*H*-spiro[isobenzofuran-1,9'-xanthen]-6'-yl)amino)-1-oxopentan-2-yl)amino)-2-oxoethyl)-4-methylpentanamide (**12**)

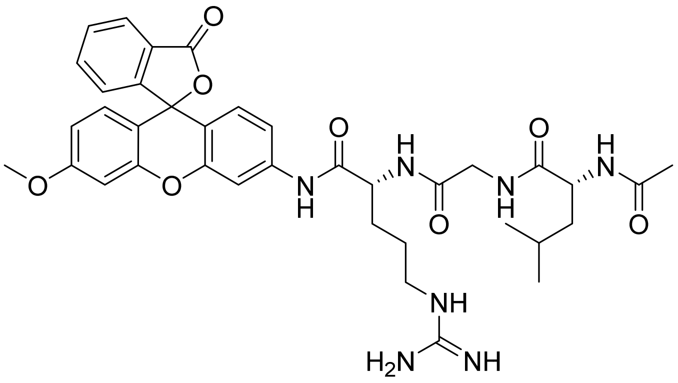

Compound **12** was synthesized from compound **10** (45 mg, 0.047 mmol) via the deprotection of Pbf using TFA (10 %, 0.8 mL) in anhydrous CH_2Cl_2 (8 mL), according to general procedure C. The residue was purified by flash column chromatography on silica gel to give compound **12** (15 mg) in 45 % yield. ^{1}H-NMR (400 MHz, DMSO-d_6) δ 10.28 (d, J = 11.9 Hz, 1H), 8.33 (q, J = 6.4 Hz, 1H), 8.08 (t, J = 7.8 Hz, 2H), 7.99 (d, J = 7.3 Hz, 1H), 7.86 (t, J = 2.7 Hz, 1H), 7.76 (t, J = 7.5 Hz, 1H), 7.70 (t, J = 7.5 Hz, 1H), 7.54 (s, 1H), 7.26-7.20 (m, 2H), 7.09 (s, 4H), 6.93 (d, J = 1.8 Hz, 1H), 6.72 (d, J = 8.7 Hz, 1H), 6.68 (d, J = 2.7 Hz, 1H), 6.65 (d, J = 8.7 Hz, 1H), 4.37 (q, J = 6.6 Hz, 1H), 4.18 (q, J = 7.3 Hz, 1H), 3.78 (s, 3H), 3.68 (t, J = 5.3 Hz, 2H), 3.07 (q, J = 6.4 Hz, 2H), 1.79 (d, J = 10.1 Hz, 3H), 1.74-1.70 (m, 1H), 1.63-1.53 (m, 2H), 1.51-1.38 (m, 4H), 0.81 (ddd, J = 17.7, 6.5, 1.5 Hz, 6H); 13C-NMR (100 MHz, DMSO-d_6) δ 173.5, 173.5, 171.4, 170.2, 169.6, 169.2, 161.7, 158.3, 157.3, 153.1, 152.3, 151.3, 141.4, 136.3, 130.8, 129.5, 129.0, 126.3, 125.3, 124.5, 116.1, 114.0, 112.6, 111.3, 106.9, 101.4, 82.6, 56.2, 55.4, 53.7, 51.9, 49.1, 42.7, 29.5, 29.4, 25.6, 24.7, 23.5, 23.0, 22.1; HRMS (ESI$^+$): m/z Calcd for $C_{37}H_{44}N_7O_8$ $[M+H]^+$: 714.3251, Found: 714.3247.

A. *Semilogarithmic Plots of Concentration-response Curves of Chemical Modification of Amino Acids*

1. Lysine specific inhibitors

(1) Anhydride inhibitors

① Acetic anhydride

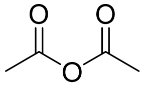

Acetic anhydride

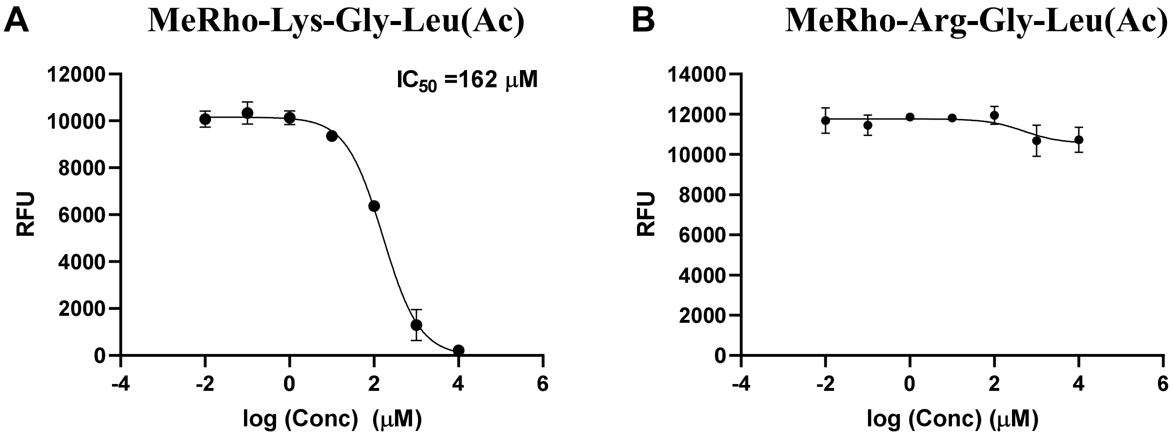

Figure S1 Amino acid blocking activity of acetic anhydride. (A) MMeRho-Lys-Gly-Leu(Ac); (B) MeRho-Arg-Gly-Leu(Ac).

② Benzoic anhydride

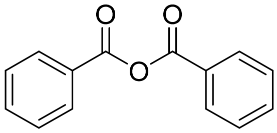

Benzoic Anhydride

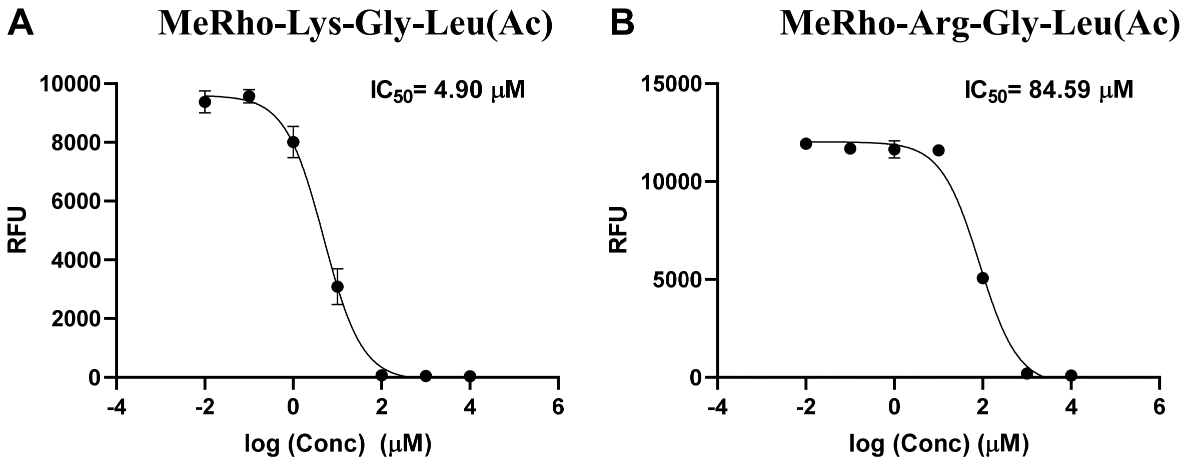

Figure S2 Amino acid blocking activity of benzoic anhydride. (A) MeRho-Lys-Gly-Leu(Ac); (B) MeRho-Arg-Gly-Leu(Ac).

③ Diethyl pyrocarbonate

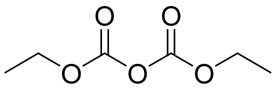

Diethyl Pyrocarbonate

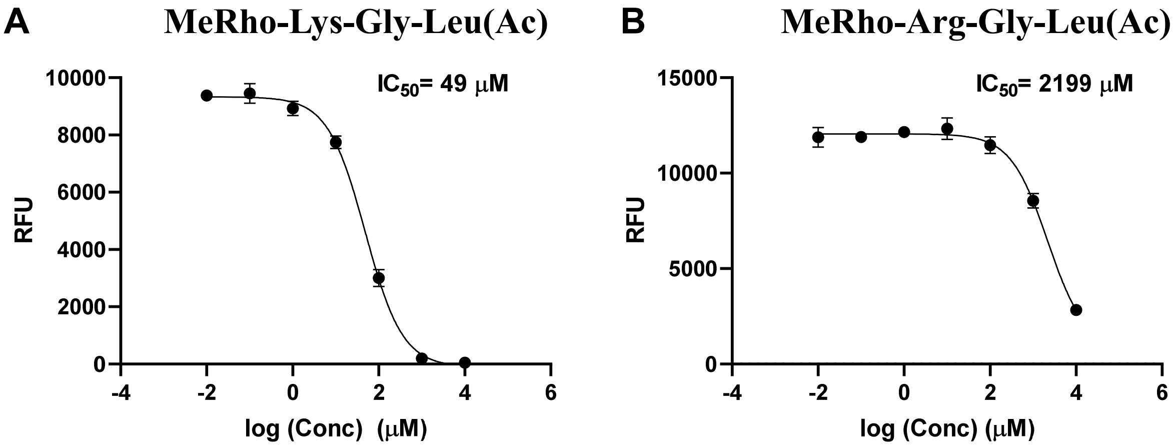

Figure S3 Amino acid blocking activity of diethyl pyrocarbonate. (A) MeRho-Lys-Gly-Leu(Ac); (B) MeRho-Arg-Gly-Leu(Ac).

④ *p*-Toluene sulfonic anhydride

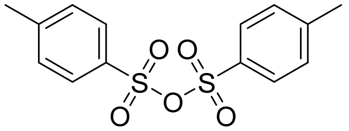

p-toluenesulfonic anhydride

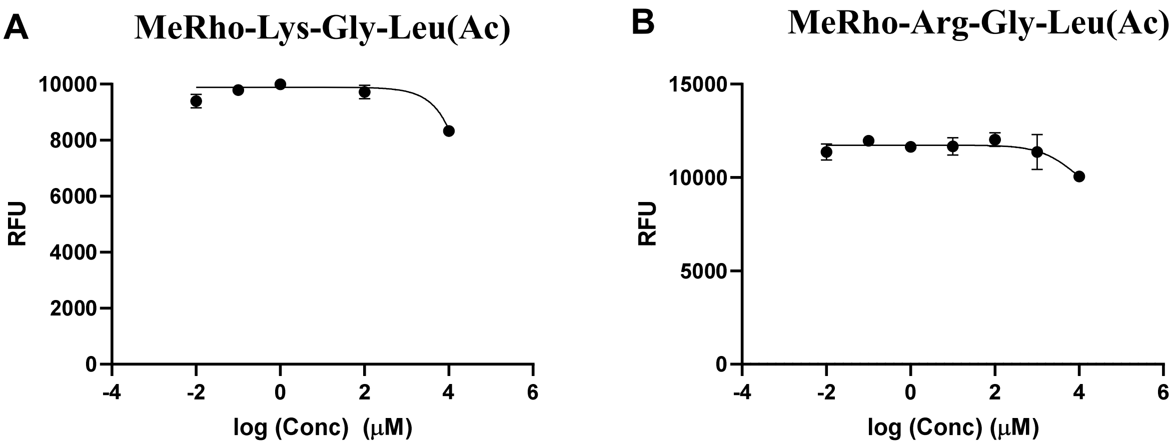

Figure S4 Amino acid blocking activity of *p*-toluene sulfonic anhydride. (A) MeRho-Lys-Gly-Leu(Ac); (B) MeRho-Arg-Gly-Leu(Ac).

(2) Cyclic anhydride inhibitors

① Maleic anhydride

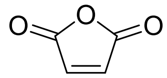

Maleic Anhydride

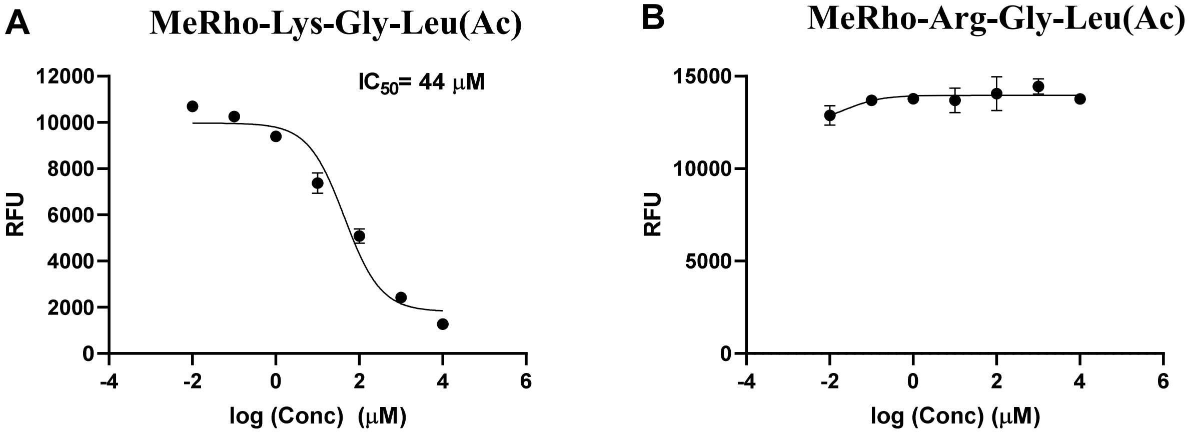

Figure S5 Amino acid blocking activity of maleic anhydride. (A) MeRho-Lys-Gly-Leu(Ac); (B) MeRho-Arg-Gly-Leu(Ac).

② Citraconic anhydride

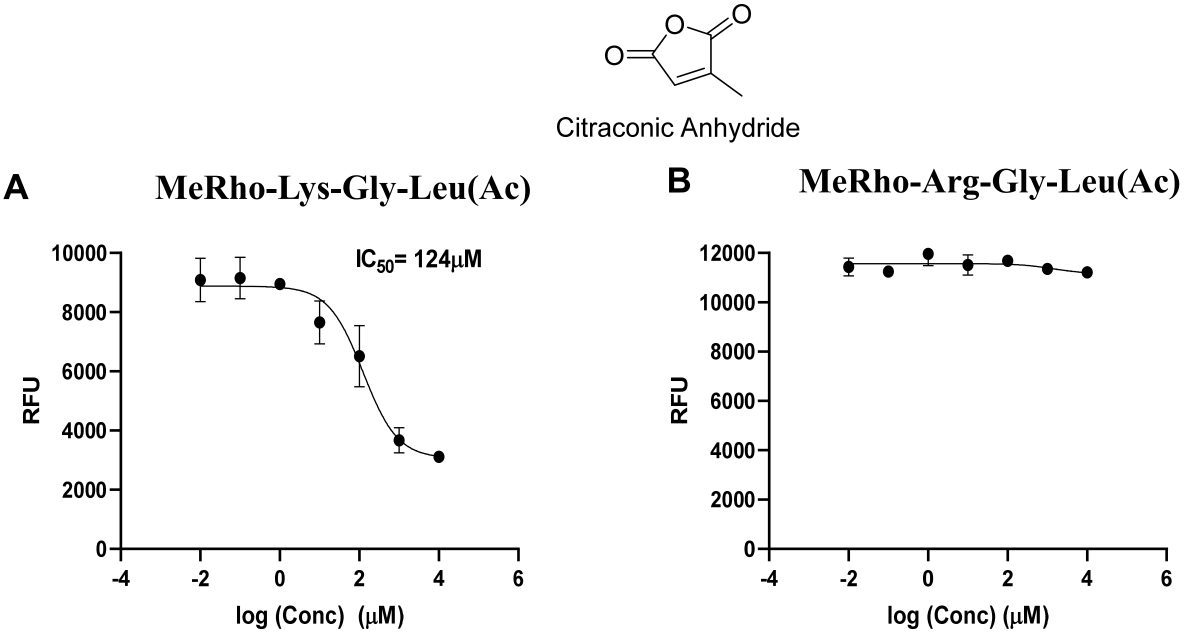

Figure S6 Amino acid blocking activity of citraconic anhydride. (A) MeRho-Lys-Gly-Leu(Ac); (B) MeRho-Arg-Gly-Leu(Ac).

③ Phthalic anhydride

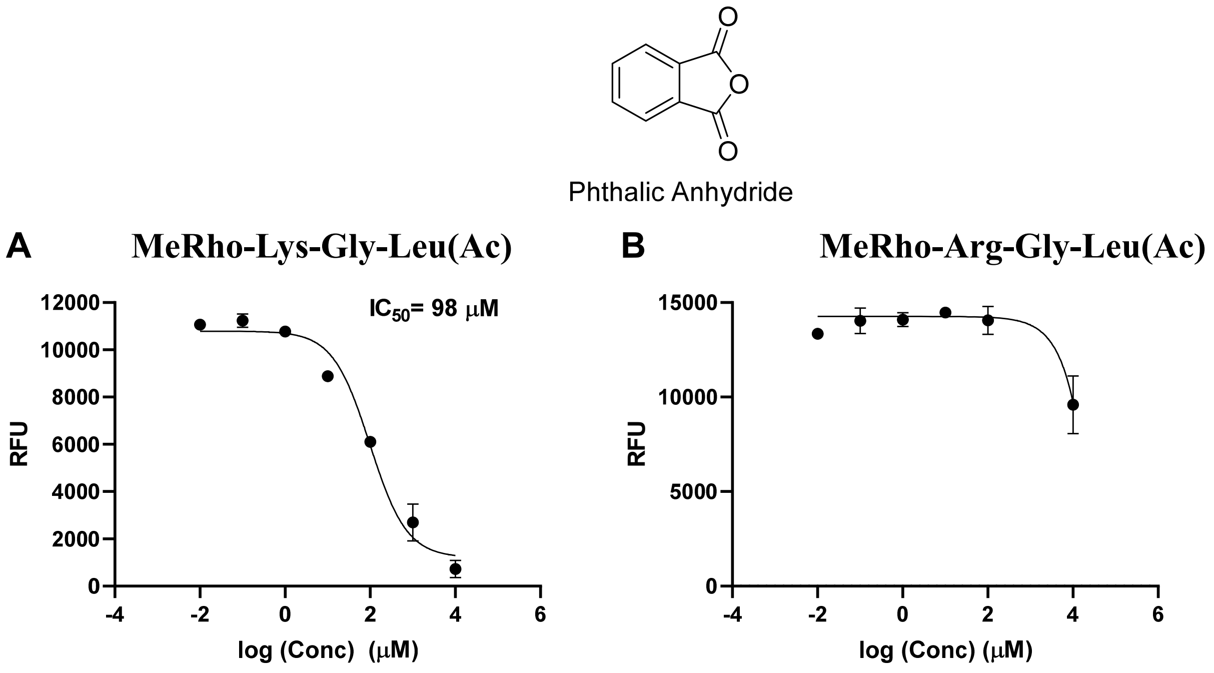

Figure S7 Amino acid blocking activity of phthalic anhydride. (A) MeRho-Lys-Gly-Leu(Ac); (B) MeRho-Arg-Gly-Leu(Ac).

2. Arginine specific inhibitors

(1) α, β-ketone aldehyde inhibitor

① Phenyl glyoxal

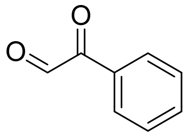

phenyl glyoxal

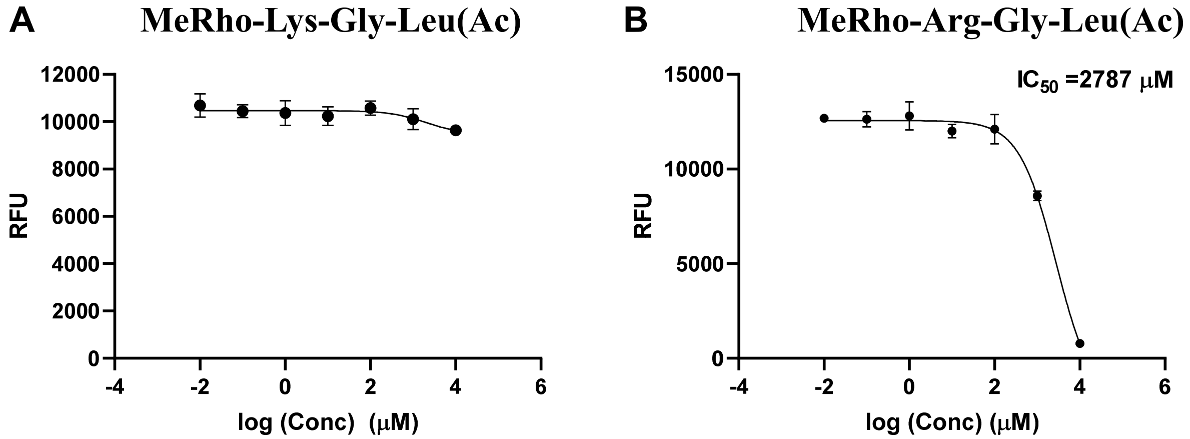

Figure S8 Amino acid blocking activity of phenyl glyoxal. (A) MeRho-Lys-Gly-Leu(Ac); (B) MeRho-Arg-Gly-Leu(Ac).

② 4-(Trifluoromethyl)phenyl glyoxal

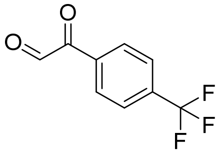

4-(trifluoromethyl)phenylglyoxal

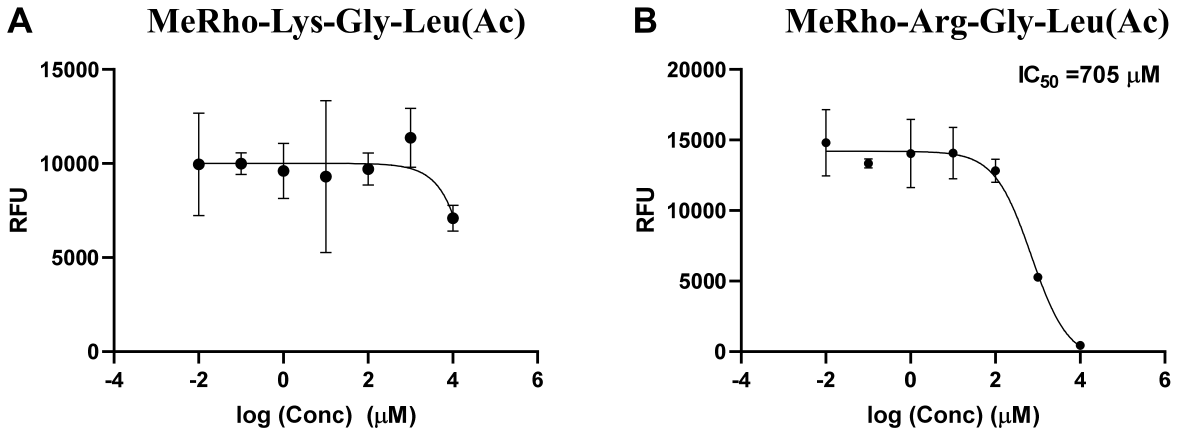

Figure S9 Amino acid blocking activity of 4-(trifluoromethyl)phenyl glyoxal. (A) MeRho-Lys-Gly-Leu(Ac); (B) MeRho-Arg-Gly-Leu(Ac).

③ 2-(Trifluoromethyl)phenyl glyoxal

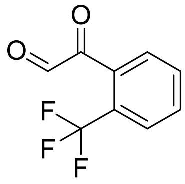

2-(Trifluoromethyl)phenyl glyoxal

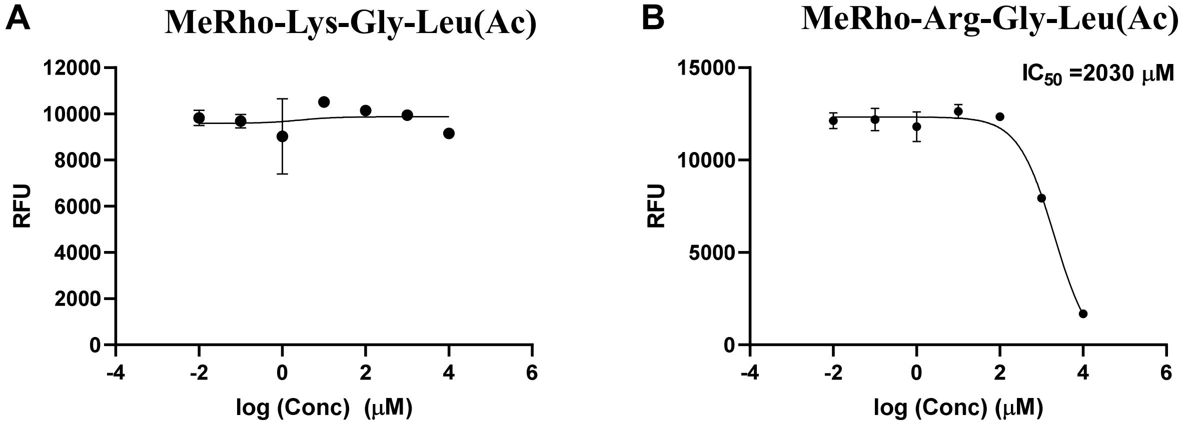

Figure S10 Amino acid blocking activity of 2-(trifluoromethyl)phenyl glyoxal. (A) MeRho-Lys-Gly-Leu(Ac); (B) MeRho-Arg-Gly-Leu(Ac).

④ 4-Nitro phenyl glyoxal

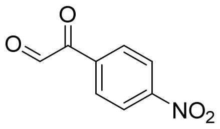

4-Nitro phenylglyoxal

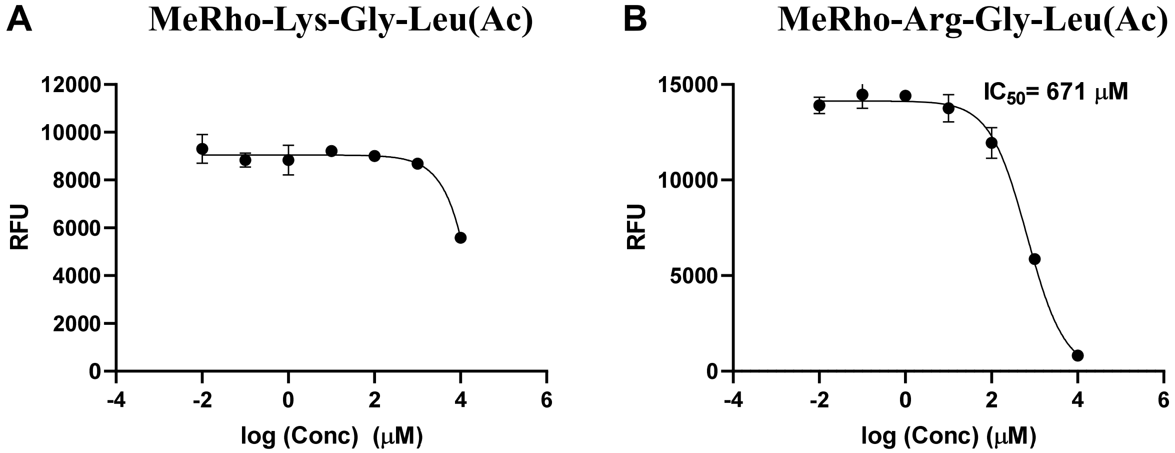

Figure S11 Amino acid blocking activity of 4-nitro phenyl glyoxal. (A) MeRho-Lys-Gly-Leu(Ac); (B) MeRho-Arg-Gly-Leu(Ac).

⑤ 4-Methoxy phenyl glyoxal

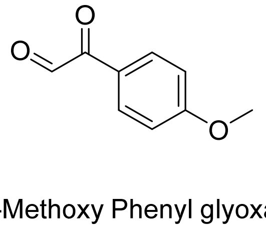

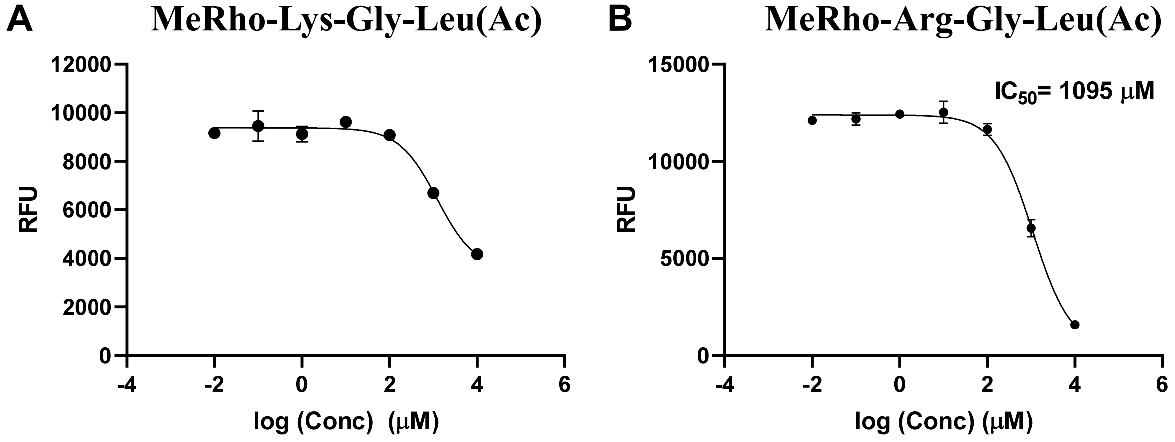

Figure S12 Amino acid blocking activity of 4-methoxy phenyl glyoxal. (A) MeRho-Lys-Gly-Leu(Ac); (B) MeRho-Arg-Gly-Leu(Ac).

⑥ 6-Methoxy-2-naphthyl glyoxal

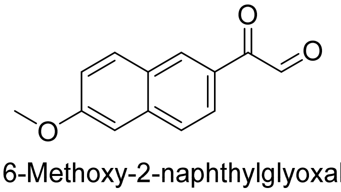

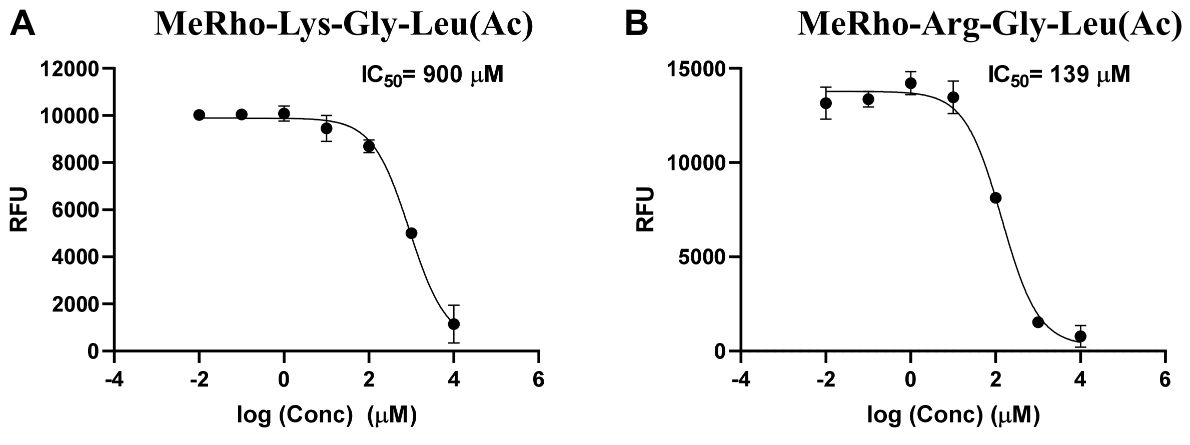

Figure S13 Amino acid blocking activity of 6-methoxy-2-naphthyl glyoxal. (A) MeRho-Lys-Gly-Leu(Ac); (B) MeRho-Arg-Gly-Leu(Ac).

(2) Monoaldehyde inhibitor

① Benzaldehyde

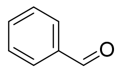

Benzaldehyde

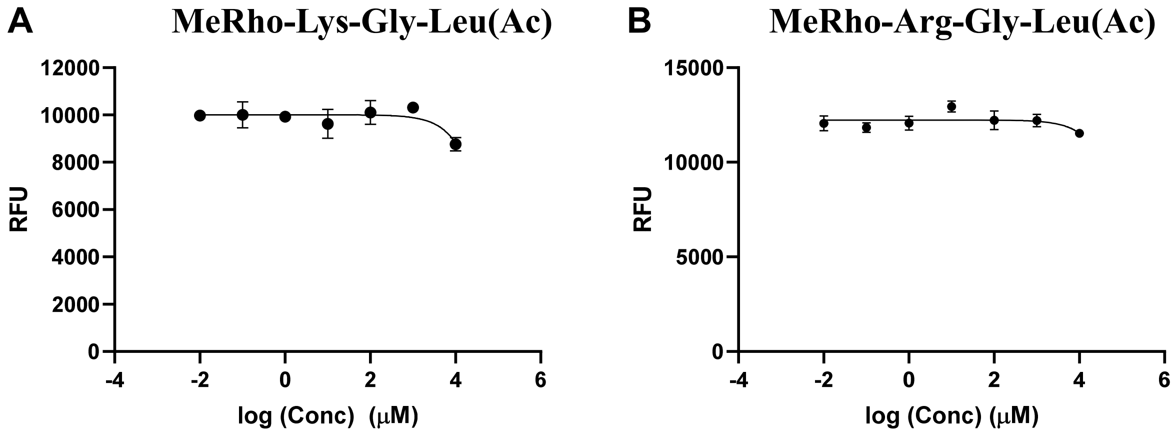

Figure S14 Amino acid blocking activity of benzaldehyde. (A) MeRho-Lys-Gly-Leu(Ac); (B) MeRho-Arg-Gly-Leu(Ac).

(3) α, β-Diketone

① 1,2 Cyclohexadione

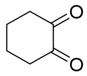

1,2 cyclohexadione

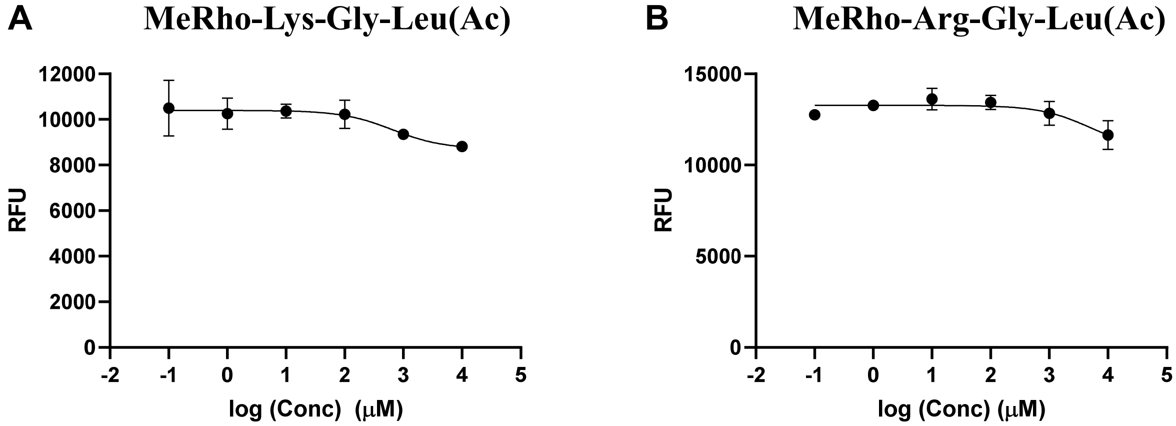

Figure S15 Amino acid blocking activity of 1,2 cyclohexadione. (A) MeRho-Lys-Gly-Leu(Ac); (B) MeRho-Arg-Gly-Leu(Ac).

② 2,3-Butadione

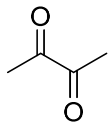

2.3-Butanedione

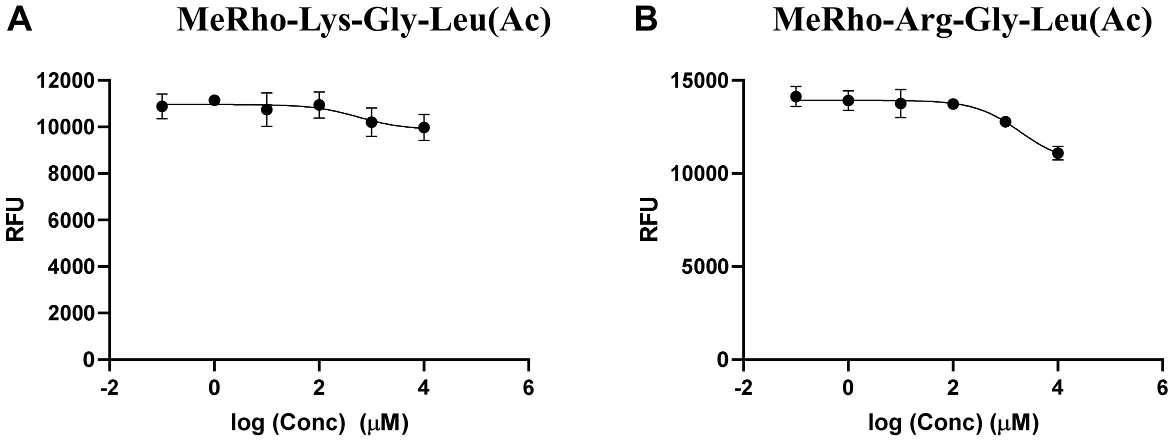

Figure S16 Amino acid blocking activity of 2,3-butadione. (A) MeRho-Lys-Gly-Leu(Ac); (B) MeRho-Arg-Gly-Leu(Ac).
